# Interindividual Variability in Duration of Action of Rocuronium in Paediatric Patients (DurAct): A Prospective Observational Study

**DOI:** 10.3390/children13010105

**Published:** 2026-01-11

**Authors:** Katerina Szturzova, Hana Zelinkova, Lenka Knoppova, Michaela Toukalkova, Tereza Kramplova, Marek Kovar, Jozef Klucka, Petr Stourac

**Affiliations:** 1 Department of Paediatric Anaesthesiology and Intensive Care Medicine, University Hospital Brno and Faculty of Medicine, Masaryk University, 625 00 Brno, Czech Republic; 2 Institute of Biostatistics and Analyses, Faculty of Medicine, Masaryk University, 625 00 Brno, Czech Republic; 3 Department of Simulation Medicine, Faculty of Medicine, Masaryk University, 625 00 Brno, Czech Republic

**Keywords:** general anaesthesia, surgery, non-depolarizing neuromuscular blockers, rocuronium, sugammadex, train of four

## Abstract

**Highlights:**

**What are the main findings?**

- Time to return of TOF 1 did not differ significantly across paediatric age groups.
- No association between TOF recovery time and sex, weight, height, or body temperature was found.

**What are the implications of the main findings?**

- The observed high interindividual variability in rocuronium duration, unrelated to patient factors, calls for individualized dosing and strict neuromuscular monitoring in paediatric patients.

**Abstract:**

**Background**: In adult patients, rocuronium shows interindividual variability related to weight, sex, and age, but paediatric data are limited. This study aimed to evaluate clinical factors influencing the duration of action of rocuronium in children. **Methods**: Patients aged between 0 and 18 years undergoing planned general anaesthesia were eligible. The primary objective was to compare the duration of clinical action of rocuronium after a single dose, measured until the return of TOF (Train of Four) 1, across three-group age categories. Secondary objectives explored the relationship between TOF recovery and sex, weight, height, initial body temperature, as well as the occurrence of postoperative complications related to general anaesthesia. **Results**: Among 96 analysed patients, no clinically relevant association was found between the duration of rocuronium action and the studied clinical factors. TOF 1 occurred at 18.1 ± 9.5 min in those aged 1 to 5 years (n = 26), 16.8 ± 14.5 min in those aged 6 to 10 years (n = 33), and 18.2 ± 13.8 min in those aged 10 to 17 years (n = 37), *p* = 0.626. A post hoc analysis revealed high variability in recovery times across TOF levels, both in the per-protocol population (e.g., TOF 1: 17.7 ± 12.9 min) and in patients who did not receive sugammadex. **Conclusions**: In paediatric patients, the duration of rocuronium action after a single dose demonstrated substantial interindividual variability, which was not explained by age, sex, weight, height, or body temperature.

## 1. Introduction

Introduced to clinical practice more than 80 years ago, muscle relaxants represent an important part of general anaesthesia in adult and paediatric patients [1]. Their role is to facilitate intubation and enable some surgical interventions in both elective and emergent settings. Compared with depolarizing agents, non-depolarizing neuromuscular blockers, including benzylisoquinolinium and aminosteroidal compounds, provide more stable relaxation with fewer postoperative complications, including reduced muscle pain and a lower incidence of hyperkalemia or malignant hyperthermia [2].

Rocuronium, a widely used synthetic aminosteroid, has an intermediate duration of action and a rapid onset. It is primarily dependent on hepatobiliary elimination, with partial renal excretion as well [2,3]. A key advantage of rocuronium is the availability of sugammadex, a specific reversal agent, which has substantially improved the safety of neuromuscular blockade management. Nevertheless, rocuronium is known to exhibit interindividual variability in duration of action in adult patients, influenced by factors such as weight, sex, and age [3,4,5]. However, pharmacodynamic data is limited in the paediatric population.

In a recent prospective observational cohort trial, a high incidence of residual neuromuscular block was observed among 291 paediatric patients in both the operating room (48.2%) and post-anaesthesia care unit (26.9%) after receiving neuromuscular blocking agents during surgery [6]. These findings highlight the need for quantitative neuromuscular monitoring in paediatric anaesthesia to detect and manage residual neuromuscular block which is crucial for patient safety.

At our workplace, monitoring of the depth of neuromuscular blockade is a standard procedure. In the present study, we hypothesized that the duration of action of single dose rocuronium depends on demographic and clinical parameters of paediatric patients. A better understanding of these factors may help optimize dosing and improve the safety of neuromuscular blockade in children.

## 2. Materials and Methods

### 2.1. Study Design and Eligibility Criteria

The DurAct study was a prospective, observational, open-label trial conducted at one site between September 2022 and August 2023. Eligible participants were paediatric patients aged 0 to 18 years admitted for a planned intervention in general anaesthesia with orotracheal or nasotracheal intubation and use of the muscle relaxant rocuronium. The American Society of Anaesthesiologists (ASA) score of 1 or 2 was allowed. Exclusion criteria included: patients requiring more types of muscle relaxants during general anaesthesia induction; patients receiving regional anaesthesia; patients requiring a rapid sequence induction of general anaesthesia; neuromuscular disease; concomitant medication interfering with the action of muscle relaxants (anticonvulsants, aminoglycosides, and polypeptide antibiotics); renal or hepatic disorders; and ASA score 3 or 4. The study was registered at www.clinicaltrials.gov (NCT05529420). The Strengthening the Reporting of Observational Studies in Epidemiology (STROBE) statement for observational studies was followed [7].

### 2.2. Procedures

In paediatric patients undergoing planned general anaesthesia with non-depolarizing muscle relaxants, induction of anaesthesia involved either standard intravenous (an opioid, anaesthetic, and muscle relaxant) or inhalational agents according to the anaesthesiologist’s preference. Anaesthesia was maintained with sevoflurane, supplemented with sufentanil or alfentanil if necessary. Rocuronium was given as a bolus and immediately flushed to ensure complete delivery. Standard monitoring during general anaesthesia was carried out, including accelerometric measurement of the depth of neuromuscular blockade (Train of Four, TOF, Avance CS2 machine, GE Healthcare, Chicago, IL, USA, relaxometry module, stimulating current of 50 mA) every minute. Stimulation was initiated immediately after induction of anaesthesia, with a stimulation frequency of 2 Hz. Electrodes were placed over the ulnar nerve at the wrist, and the thumb was left free to move for accurate accelerometric recording. Baseline calibration of the device was performed by trained technicians. Normalization of the TOF ratio was confirmed prior to rocuronium administration. Available in two sizes to accommodate smaller hands, the sensor was placed while the child was still awake and able to cooperate. A second portable device (TOF-watch SX, Organon Teknika, Jersey City, NJ, USA) for contralateral TOF measurement was available for use if no sufficient TOF decrease was observed. The forearm and hand were supported and the sensor and electrodes secured to minimize movement artefacts; the thumb was allowed to move freely for accelerometry. Continuous body temperature monitoring was also carried out, with temperature recorded whenever TOF values changed. Subsequently, these parameters, comprising the age-related minimum alveolar concentration (MAC) of sevoflurane, were collected on paper-based case report forms (CRF). Sevoflurane can potentiate the effects of neuromuscular blockers; therefore, MAC, as a standardized measure of anaesthetic depth, was recorded to assess its potential contribution to variability in rocuronium duration.

Rocuronium was administered at a dose of 0.6 mg/kg. Patient demographic and clinical parameters, dose of rocuronium, time of its administration, and time of TOF 1 measurement were documented in the CRF. The time to TOF 1 was defined as the interval until the first twitch reappeared following ulnar nerve stimulation, measured as contraction of the adductor pollicis muscle. In patients requiring further doses of rocuronium, the TOF intervals following administration of these additional doses were not included in the analyses. If there was no need to add further doses of the muscle relaxant, TOF 2, TOF 3, TOF 4, and TOF-ratio ≥ 0.9 were also registered in addition to TOF 1. TOF-ratio ≥ 0.9 signifies the time to full recovery from the non-depolarising muscle relaxant’s effect. The TOF stimulation pattern was chosen to ensure precise detection of changes in TOF responses. In addition, the reversal of neuromuscular blockade by sugammadex, if applicable, and the Aldrete score, a system used to assess the recovery of patients from anaesthesia, in the first and fifth minutes after extubation were recorded [8].

In patients monitored in a recovery room after an intervention, possible complications occurring within at least 30 min postoperatively (desaturation, the need for oxygen therapy, tachycardia, bradycardia, aspiration, sore throat, cough, and hoarseness) and the Aldrete score at the 30th minute were documented. Tachycardia and bradycardia were defined using age-specific heart rate thresholds (i.e., tachycardia: >160 bpm for infants <1 year, >140 bpm for children 1–5 years, >120 bpm for children >5 years; bradycardia: <80 bpm for infants, <70 bpm for children 1–5 years, <60 bpm for children >5 years). In case of a suspicion of aspiration pneumonia, a chest X-ray was performed within the first 24 h after the intervention.

### 2.3. Outcomes

The primary endpoint was the duration of clinical action of rocuronium after a single-dose administration, measured until the return of TOF 1. The primary analysis consisted of comparing this endpoint between three different paediatric age categories.

Secondary endpoints involved a comparison of the duration of clinical action of rocuronium after a single-dose administration, measured until the return of TOF 1, relative to patient’s sex, weight (three categories), and height (two categories); a comparison of the interval from single-dose rocuronium to TOF 2, 3, and 4 in different paediatric age categories and relative to patient’s sex, weight, and height; a comparison of the interval from single-dose rocuronium to full recovery in different paediatric age categories and relative to patient’s sex, weight, height, and initial body temperature (two categories); identification of a patient subgroup requiring antagonization after a surgical procedure; and monitoring of postoperative complications of general anaesthesia. The rationale for including the analyses of TOF 1–4 and TOF-ratio ≥ 0.9 was to provide a more detailed understanding of neuromuscular recovery dynamics and interindividual variability in the duration of action.

### 2.4. Bias

To minimize selection bias, eligibility criteria were designed to include a broad paediatric population undergoing standard anaesthetic procedures, while excluding factors known to influence neuromuscular blocker metabolism. Standardized protocols for anaesthesia induction and TOF monitoring were followed across all cases to ensure consistency. Data collection and entry were performed by trained staff, with periodic checks for accuracy.

### 2.5. Statistical Analysis

Statistical analyses were conducted to compare different patient groups. Quantitative variables (age, weight, height, and body temperature) were grouped into two or three categories to explore potential non-linear relationships and ensure balanced subgroup sizes for statistical comparisons. Parametric data were analysed using analysis of variance (ANOVA) with a post hoc Tukey–Kramer test for pairwise comparisons. Non-parametric data were analysed using the Kruskal–Wallis test followed by Dunn’s post-test. Frequency comparisons were performed using the Chi-square test. Missing data were handled by excluding affected patients. Only patients with TOF 1 data were analyzed, while missing values for other variables led to exclusion only from the respective analyses.

Results are presented as mean ± standard deviation or frequency, as appropriate. A *p*-value < 0.05 was considered statistically significant. Potential confounders, including demographic and clinical variables (e.g., age, weight, height, and initial body temperature), were considered in subgroup comparisons to account for their influence on the outcomes. The sample size was determined pragmatically based on the average annual number of paediatric surgeries at our centre using rocuronium. Statistical analyses were performed using InStat version 3.10 (GraphPad Software, Boston, MA, USA; www.graphpad.com). Sensitivity analyses were not conducted as the study primarily focused on descriptive and exploratory subgroup analyses based on predefined categories.

## 3. Results

### 3.1. Patient and Treatment Characteristics

**Figure 1:**
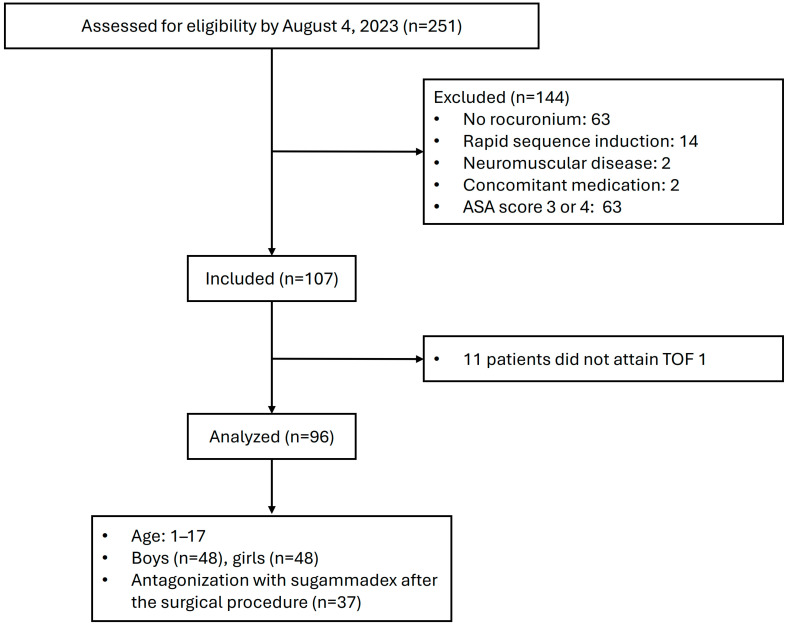
Flowchart of patient inclusion.

During the recruitment period, 107 paediatric patients were enrolled in our trial (Figure 1). No patients under 1 year of age or over 17 were recruited. Out of them, 11 patients did not attain TOF 0 and TOF 1 after the standard dose of rocuronium, as their TOF values remained higher; this finding was confirmed by the second portable TOF measurement device. No additional values from the second device were thus included in the study. Consequently, the per-protocol population comprised a total of 96 patients eligible for the outcome analysis. Patient and treatment characteristics are summarized in Table 1.

Demographic characteristics such as sex, weight, and height and clinical factors, initial body temperature, were recorded as these factors were hypothesized to influence drug metabolism and pharmacodynamics. To compare different groups of patients, we divided each of the characteristics into two to three balanced categories as follows: age in three categories (1 to 5 years, 6 to 10 years, 11 to 17 years), weight in three categories (0 to 20 kg, 21 to 40 kg, 41 to 115 kg), height in two categories (80 to 130 cm and 131 to 183 cm), and initial temperature in two categories (36.5 °C or less and 36.6 °C or more).

### 3.2. Primary Endpoint

The duration of clinical action of rocuronium until the return of TOF 1 was compared between the three age groups, with 26 (1 to 5 years: 18.1 ± 9.5 min), 33 (6 to 10 years: 16.8 ± 14.5 min), and 37 (10 to 17 years: 18.2 ± 13.8 min) patients, respectively. Neither of these comparisons showed statistically significant differences, with *p*-values of 0.985 and 0.626, respectively (Table 2).

### 3.3. Secondary Endpoints

Further correlative analyses between TOF 1, 2, 3, 4, and TOF-ratio ≥ 0.9 and patient’s sex, weight, height, and initial body temperature did not reveal any significant differences, except for the TOF-ratio ≥ 0.9 which was significantly higher in subjects with a body height of 131 cm or more relative to those with a smaller body height (77.2 ± 27.9 min versus 60.4 ± 23.9 min, *p* = 0.002). Over one third of patients (n = 37, 38.5%) required antagonization with sugammadex after the surgical procedure. Detailed results are available in Table 2.

The following postoperative complications were reported: desaturation after extubation in five patients, tachycardia in two patients, the need for oxygen therapy in nine patients, postoperative nausea and vomiting in two patients, sore throat in two patients, and hoarseness and/or cough in four patients. No cases of bradycardia with a heart rate below 50 beats per minute, aspiration, or pneumonia on x-ray imaging were reported.

Altogether, 14 patients (15%) presented with at least one complication. This included nine patients presenting with one complication, one patient with two complications, three patients with three complications, and one patient with four complications.

Among the 56 patients who did not receive sugammadex, complications were as follows: five patients experienced desaturation; one patient had tachycardia; one patient had hoarseness; one patient had both hoarseness and desaturation; one patient had both tachycardia and desaturation; and one patient experienced postoperative nausea and vomiting, hoarseness, and cough. All of these patients achieved a TOF-ratio ≥ 0.9; therefore, we consider it unlikely that the reported complications were related to residual neuromuscular blockade.

### 3.4. Post Hoc Analysis

**Figure 2:**
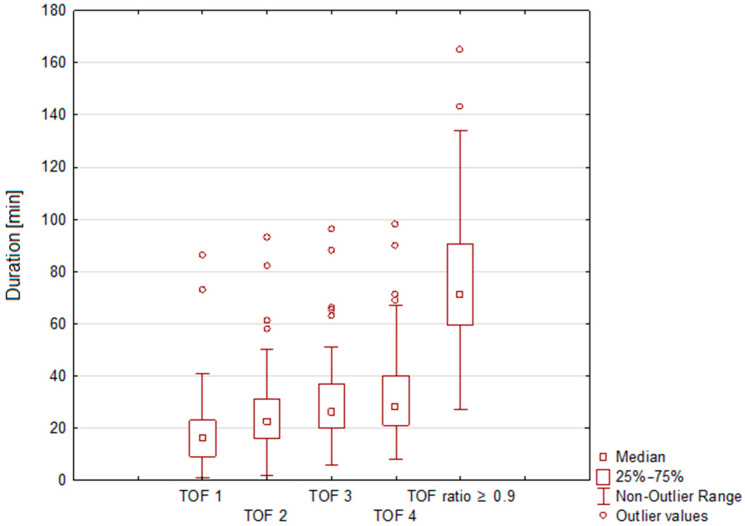
Variability of TOF 1, 2, 3, and 4 in the per-protocol population (n = 96) and TOF-ratio ≥ 0.9 in patients who did not receive sugammadex antagonization (n = 56).

In an exploratory analysis, we examined variations in TOF 1, 2, 3, and 4 in the per-protocol population and TOF-ratio ≥ 0.9 in patients who did not receive sugammadex antagonization. As illustrated in Figure 2, all TOF values demonstrated high variability, reflected by wide ranges. For example, in the per-protocol population, the mean durations for TOF 1 and 2 were 17.7 ± 12.9 min and 25.6 ± 15.1 min, respectively. More details for the per-protocol population are summarized in Table 1.

## 4. Discussion

To the best of our knowledge, this is the first prospective study exploring interindividual variability in the duration of action of rocuronium in paediatric patients. Our results did not show any consistent or clinically meaningful relationship between the duration of clinical activity after a single-dose administration and different variables, including age, sex, weight, height, and initial body temperature among 96 analysable cases aged 1 to 17 years. However, and most interestingly, we noted high variability in TOF 1, 2, 3, 4, and TOF-ratio ≥ 0.9 in both the per-protocol population and in patients who did not receive sugammadex antagonization.

In our previous study, we did not observe any statistically significant difference in the mean time to TOF 1–2 between deep and intermediate neuromuscular blockades [9]. These results indirectly demonstrate that the duration of action of muscle relaxants does not depend on the depth of neuromuscular blockade. Two other randomized trials evaluated the impact of different neuromuscular blockades on surgical conditions in children, but the investigators either used continuous infusions of rocuronium or did not provide correlative analyses similar to ours [10,11].

In 2012, the results of a phase III multicentre study were published, investigating the time from the end of rocuronium administration at three different doses (0.45 mg/kg, 0.6 mg/kg, and 1.0 mg/kg) to the return of TOF3 in 207 paediatric patients aged 0 to 17 years, who underwent inhalational general anaesthesia between 2004 and 2007. The median time to TOF3 showed high variability, ranging from 21 to 114 min. It was longer in the higher dose groups regardless of age, and also prolonged in neonates and infants compared to older children [12]. In a systematic review by Vanlinthout et al., data from 71 controlled trials involving 4319 participants confirmed significant variability in the duration of neuromuscular blockade. Recovery times were longer in neonates and infants compared to older children, and were further prolonged by the use of volatile anaesthetics such as sevoflurane and isoflurane [13]. In our study, no infants were included, partly because general anaesthesia is less common in this age group and partly due to chance, as infants treated during the study period were not encountered by the investigators.

Previous studies in the adult population have shown different results, with longer durations of rocuronium action observed in patients with renal or liver disease, in obese individuals, females, older patients, and those with reduced portal venous blood flow during laparoscopic surgery with CO_2_ pneumoperitoneum [3,4,5,14,15,16]. Varrique found that age-related reductions in renal function led to altered drug disposition in elderly patients, resulting in differences in the pharmacokinetic and pharmacodynamic properties of rocuronium. These changes were primarily due to reduced clearance and increased drug exposure, rather than altered sensitivity to the drug [5]. Xue et al. concluded that sensitivity to rocuronium was 30% lower in men than women, suggesting the necessity of routine dose reduction in female patients [4]. Another noteworthy observation came from a working group led by Arain, who compared the duration of action of cisatracurim with equipotent doses of vecuronium and rocuronium in older patients, showing significantly higher variability with the latter two compounds [17]. Last but not least, Ahlström and colleagues presented the results of the first pharmacogenomic study in almost one thousand women operated on for breast cancer. Genetic variation in the SLCO1A2 gene, which encodes the uptake transporter Organic Anion Transporting Polypeptide 1A2 (OATP1A2), explained 4% of the variability in rocuronium metabolism [18]. These genetic variations have been shown to be influenced by ethnicity [19]; however, this was unlikely to affect our results, as all participants in our cohort were of Caucasian descent.

Our study did not reflect observations in the adult population, probably due to several reasons. First, the age span in children (until 18 years of age) is considerable smaller than in adults. Second, sex-related differences are clearly pronounced in the adults and less so in children, particularly those under 13 years of age. Moreover, further factors could have impacted the outcomes, comprising drug interactions and genetically based variability in drug absorption, distribution, metabolism (linked to polymorphisms of cytochrome P450), and excretion [20].

The clinical implications of our findings are substantial. The high variability in the duration of action of rocuronium underscores the importance of individualized dosing and rigorous neuromuscular monitoring in paediatric anaesthesia to ensure patient safety. The strengths of our study include the prospective design and rigorous conduct at a single centre. Although the single-centre design may limit generalizability, this setup reduced variability in clinical practices and ensured adherence to uniform monitoring protocols. We attempted to address potential confounders, such as demographic and clinical parameters, but unmeasured factors (e.g., genetic polymorphisms, comorbidities, differences in muscle mass and liver size relative to body weight) could still have influenced the outcomes. Observer bias was minimized by standardizing data documentation and analysis procedures. Moreover, a larger sample size could have allowed the detection of more subtle differences with possible hypothesis-generating implications.

In our study, all patients received sevoflurane for anaesthesia maintenance, which may have prolonged the duration of rocuronium action. Nevertheless, because this exposure was consistent across the cohort, we consider our analyses valid. Moreover, we believe that a brief inhalational induction with sevoflurane is unlikely to have had a meaningful impact on recovery times. The minimum alveolar concentration was monitored in all patients throughout anaesthesia (Table 1). Another limitation of our study is that the values for TOF-ratio ≥ 0.9 were reported as non-normalized. This may have influenced the precise determination of recovery to TOF-ratio ≥ 0.9, and we acknowledge that normalization would provide a more accurate assessment of neuromuscular recovery. Finally, as no formal sample size calculation was performed, the study may have been underpowered to detect small-to-moderate effect sizes, and the possibility of type II error cannot be excluded. Non-significant subgroup comparisons should therefore be interpreted with caution and regarded as exploratory. In line with this, our findings should not be interpreted as definitive evidence of absence of an effect of demographic or clinical factors, but rather as an indication that no large effect was detectable within the limits of this cohort.

## 5. Conclusions

In conclusion, we have demonstrated a substantial variability in the duration of clinical activity after a single dose of rocuronium in paediatric patients, which could not be explained by differences in age, sex, weight, height, or initial body temperature. This information is crucial for patient safety and is in line with the previously reported observation of a high incidence of residual neuromuscular block in paediatric patients [6]. Factors, such as hepatic enzyme polymorphisms of cytochrome P450 and potential genomic correlations, hold great potential for finding causal relationships explaining the observed variability.

## Figures and Tables

**Table 1:** Baseline characteristics and measured variables in the patient cohort. Weight, height, and age are expressed as median (interquartile range), while the remaining parameters are presented as mean ± standard deviation with 95% confidence intervals or as frequency in percentage.

Parameter	Value
1. Demographic and Clinical Data	
Total number of analysed patients	96
Sex (male, female)	48 (50%), 48 (50%)
Weight (kg)	30 (14.0–75.3)
Height (cm)	136 (96.9–175.7)
Age (years)	8.5 (2.9–16.1)
Initial temperature (°C)	36.5 (0.4)
2. Type of Surgery	
General surgery	10 (10.4%)
Ear, nose and throat surgery	85 (88.5%)
Plastic surgery	1 (1.0%)
3. Anesthesia Management	
Induction	
Inhalation	12 (12.5%)
Intravenous	84 (87.5%)
Sufentanil (µg/kg)	0.2 (0.1)
Alfentanil (mg/kg)	0.02
Propofol (mg/kg)	3.2 (0.7)
Rocuronium (mg/kg)	0.6 (0.1)
4. Neuromuscular Monitoring and Sevoflurane MAC	
TOF 1 (min)	17.7 (12.9)
MAC of sevofluran (%)	1.1 (0.3)
TOF 2 (min)	25.6 (15.1)
MAC of sevofluran (%)	1.1 (0.2)
TOF 3 (min)	30.0 (16.0)
MAC of sevofluran (%)	1.1 (0.3)
TOF 4 (min)	32.3 (16.3)
MAC of sevofluran (%)	1.1 (0.3)
TOF-ratio ≥ 0.9	70.0 (27.2)
MAC of sevofluran (%)	0.6 (0.4)
5. Reversal and Recovery	
Reversal	
Yes	37 (38.5%)
No	56 (58.3%)
Sugammadex (mg/kg)	2.1 (0.5)
Aldrete score at 1 min	7.2 (1.2)
Aldrete score at 5 min	7.7 (1.4)
Aldrete score at 30 min	8.4 (1.6)

Abbreviations: TOF, train of four; MAC, minimal alveolar concentration.

**Table 2:** The duration of clinical action of rocuronium until the return of TOF 1, TOF 2, TOF 3, TOF 4, and TOF-ratio ≥ 0.9 with respect to patient’s sex, weight, height, age, and initial temperature.

Parameter	Categories	N	TOF 1		TOF 2		TOF 3		TOF 4		TOF-Ratio ≥ 0.9	
			Mean ± SD	*p*-Value	Mean ± SD	*p*-Value	Mean ± SD	*p*-Value	Mean ± SD	*p*-Value	Mean ± SD	*p*-Value
All patients	N/A	96	17.7 ± 12.9		25.6 ± 15.1		30.0 ± 16.0		32.3 ± 16.3		70.0 ± 27.2	
Sex	female	48	18.1 ± 12.1	0.684	26. 2 ± 14.6	0.710	30.2 ± 15.7	0.956	33 ± 16.5	0.797	72.8 ± 29.0	0.444
	male	48	17.3 ± 13.8		25.0 ± 15.8		29.7 ± 16.3		31.5 ± 16.2		67.3 ± 25.5	
Age (3 cat.) [years]	1 to 5	26	18.1 ± 9.5	0.626	24.6 ± 11.9	0.657	28.3 ± 12.6	0.709	33.3 ± 14.3	0.703	64.7 ± 28.3	0.228
	6 to 10	33	16.8 ± 14.5		24. 2 ± 16.2		28.7 ± 16.7		30.4 ± 16.1		68.8 ± 27.3	
	11 to 17	37	18.2 ± 13.8		27.3 ± 16.6		32.1 ± 17.6		33.1 ± 17.9		74.7 ± 26.4	
Weight (3 cat.) [kg]	0 to 20	25	17.3 ± 9.5	0.288	23.8 ± 12.1	0.146	27.3 ± 12.9	0.142	32.3 ± 14.8	0.233	63.5 ± 29.6	0.052
	21 to 40	40	16 ± 12.9		22.9 ± 15.0		27. 6 ± 15.3		29.2 ± 15.3		68.2 ± 26.7	
	41 to 115	31	20.3 ± 15.1		30.0 ± 16.9		35.0 ± 18.1		36.1 ± 18.2		77.5 ± 25.1	
Height [cm]	80 to 130	39	17.0 ± 8.4	0.706	22.7 ± 10.0	0.485	26.8 ± 10.7	0.317	30.4 ± 12.3	0.748	60.4 ± 23.9	0.002
	131 to 183	52	18.2 ± 15.6		26. 9 ± 18.0		32.2 ± 19.1		33.1 ± 18.9		77.2 ± 27.9	
Temperature (2 cat.) [°C]	≤36.5 °C	50	17.1 ± 8.8	0.683	24.3 ± 10.3	0.910	28.3 ± 11.4	0.964	31.4 ± 12.5	0.737	68.1 ± 26.2	0.520
	≥36.6 °C	46	18.4 ± 16.3		26.9 ± 19.0		31.8 ± 19.8		33.2 ± 19.6		72.0 ± 28.6	

Abbreviations: cat., category; TOF, train-of-four; SD, standard deviation with 95% confidence intervals.

## Data Availability

The original contributions presented in this study are included in the article. Further inquiries can be directed to the corresponding author.

## Abbreviations

The following abbreviations are used in this manuscript:

TOF	Train of Four
ASA	American Society of Anaesthesiologists
STROBE	Strengthening the Reporting of Observational Studies in Epidemiology
CRF	case report forms
ANOVA	analysis of variance
